# Drivers of desert plant beta diversity on the Qinghai–Tibet plateau

**DOI:** 10.1002/ece3.10993

**Published:** 2024-02-20

**Authors:** Lu Wen, Kexuan Zhao, Haoyu Sun, Gang Feng, Qiang Sun, Cunzhu Liang, Zhiyong Li, Lixin Wang, Jens‐Christian Svenning

**Affiliations:** ^1^ Ministry of Education Key Laboratory of Ecology and Resource Use of the Mongolia Plateau, Collaborative Innovation Center for Grassland Ecological Security, School of Ecology and Environment Inner Mongolia University Hohhot China; ^2^ Center for Ecological Dynamics in a Novel Biosphere (ECONOVO) & Center for Biodiversity Dynamics in a Changing World (BIOCHANGE), Department of Biology Aarhus University Aarhus C Denmark

**Keywords:** beta diversity, desert plants, environmental influence, Qinghai–Tibet plateau

## Abstract

The desert ecosystem of the Qinghai–Tibet Plateau (QTP) is an important component of China's desert ecosystem. Studying the mechanisms shaping the taxonomic, phylogenetic, and functional beta diversity of plant communities in the QTP desert will help us to promote scientific conservation and management of the region's biodiversity. This study investigated the effects of environmental (including altitude, climate factors, and soil factors) and geographic distances on three facets of beta diversity as well as their turnover and nestedness components based on field survey data. The results showed that turnover components dominate the three facets of beta diversity. However, the turnover contributions to phylogenetic and functional beta diversity were lower than for taxonomic beta diversity. Environmental distance had a greater influence than geographic distance, with the former uniquely explaining 15.2%–22.8% of beta diversity and the latter explaining only 1.7%–2.4%. Additionally, the explanatory power of different factors for beta diversity differed between herbs and shrubs, with environmental distance being more important for the latter. Distance‐based redundancy analysis suggested that soil total potassium content had a substantial impact on the beta diversity of three dimensions, with mean temperature of the coldest month and soil total phosphorus content having a substantial impact on taxonomic and functional beta diversity as well. Our results support that environmental sorting plays a predominant role in shaping plant community composition across QTP desert ecosystems. To maintain the plant diversity of this region, it is crucial to prioritize the conservation of its diverse environmental conditions and actively mitigate its degradation by anthropogenic pressures.

## Graphical abstract

The study investigated the beta diversity of plant communities in the Qinghai–Tibet Plateau desert, revealing that turnover components predominantly shape beta diversity. Environmental distance exerted a greater influence than geographic distance, with soil factors, such as total potassium content, significantly impacting beta diversity. The findings underscore the crucial role of environmental sorting in shaping plant communities and emphasize the importance of conserving diverse environmental conditions to maintain the region's plant diversity.

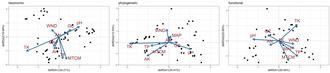

## 1 INTRODUCTION

Beta diversity refers to the variation in species composition of biological communities along environmental gradients (Whittaker, 1972; Whittaker & Niering, 1975) or, more generally, in space or time (Graham & Fine, 2008; Socolar et al., 2016), and can be partitioned into separate turnover and nestedness components (Harrison et al., 1992; Lennon et al., 2001; Matthews et al., 2019; Wang et al., 2011). Turnover represents a change in species composition due to the replacement of species, while nestedness indicates a pattern where smaller species assemblages are subsets of larger ones, reflecting the gain or loss of certain species (Baselga, 2010). The addition of species through turnover enhances gamma diversity, while a nested pattern limits it (Baselga, 2010). In nature, the proportion of turnover and nestedness in communities varies among geographic regions and ecosystem types (Baselga, 2010; Carvalho et al., 2012).

Plant beta diversity is determined by environmental and spatial factors—notably dispersal—together, with the relative influences of these factors varying across habitat types, different scale and growth forms (Bernard‐Verdier et al., 2013; Myers et al., 2013; Steinitz et al., 2006; Wang et al., 2019, 2021). For instance, in a dryland ecosystem, climate exhibited a significant influence on herb beta diversity, while both soil and topographic factors explained a greater proportion of the variation in shrub beta diversity (Wang et al., 2021). In an alpine ecosystem, environmental factors were shown to be more important than geographic distance for sharping the pattern of beta diversity by most of the studies (Hu et al., 2022; Malanson et al., 2022; Zellweger et al., 2017). However, it was also found that phylogenetic beta diversity patterns were shaped mainly by dispersal constraints (Hu et al., 2023). Investigating the patterns and determine factors of beta diversity can provide important insights into the mechanisms underlying the maintenance of biodiversity and offer useful information for biodiversity conservation and ecosystem management (Deli et al., 2023; Socolar et al., 2016).

Biodiversity is a multifaceted concept that encompasses a wide range of biological variations (Si et al., 2016; Swenson et al., 2012). Taxonomic beta diversity has been used as a surrogate for compositional differences between communities (Whittaker, 1960). Phylogenetic beta diversity takes the evolutionary relationships into account, reflecting species evolutionary history (Cardoso et al., 2014; Graham & Fine, 2008). Functional beta diversity emphasizes the overall differences in traits among species (Petchey & Gaston, 2002, 2006). Therefore, by considering the patterns and factors influencing multifaceted beta diversity, we can gain a more comprehensive understanding of the mechanisms that shape community assembly and ecosystem functioning.

Desert ecosystems, encompassing 20%–30% of the Earth's land area (Hadley & Szarek, 1981), are often wrongly perceived as barren and inhospitable. Instead, they actually support a surprisingly great diversity, including some unique species that have evolved specialized adaptations to thrive in the challenging desert environment (Wen et al., 2014; Whitford & Duval, 2019). Moreover, in recent years, global warming has resulted in an elevated frequency of extreme weather events (Cai et al., 2014; Meehl et al., 2000). Specifically, in desert regions, droughts are anticipated to intensify in the future, which threatens the plant diversity (Lindh et al., 2014; Tang et al., 2018). Therefore, by exploring the mechanisms underlying plant community assembly patterns in deserts, we can gain valuable insights to better predict how deserts will respond to global change. Although several studies have investigated beta diversity in arid or desert regions, the majority of these studies have focused on grasslands (Bergholz et al., 2017; Li et al., 2021; Tang et al., 2018) or wetlands (Hu et al., 2022; Jiang et al., 2021, 2022). As a result, there is still a substantial research gap in our understanding of the diversity of desert ecosystems (Goettsch & Hernández, 2006; Wang, Qu, et al., 2022a; Wang, Wang, et al., 2022b).

As an important part of the desert biome globally, the Qinghai–Tibet Plateau (QTP) desert ecosystem is located at the highest plateau in the world, harbors many alpine endemics and is listed among the main biodiversity hotspots of the Northern Hemisphere (Favre et al., 2015; Myers et al., 2000). Understanding the patterns, components and drivers of the three facets of beta diversity in plants in this region can help guide conservation actions, for example, in terms of spatial prioritization. Previous research has highlighted that rapid speciation and environmental filtering play a pivotal role in shaping community assembly in the QTP (Yan et al., 2013). The harsh and unique environmental conditions of the QTP desert have led to the relatively recent evolution of lineages with adaptations to survive in this cold and arid environment and subsequent adaptive radiation of species in these lineages into different parts of the region's environmental space. Previous studies have demonstrated phylogenetic and functional convergence among species in the QTP (Wang, Wang, et al., 2022b; Yan et al., 2013). Therefore, we hypothesize that taxonomic beta diversity should be substantially higher than phylogenetic and functional beta diversity and contain a dominant turnover component (H1). Furthermore, we hypothesize that the beta diversity in this region is more correlated with environmental distance than geographic distance, indicating a predominant role of environmental sorting (H2). Finally, we expect that soil factors should have a greater impact on beta diversity than climatic factors (H3), due to the high edaphic heterogeneity in the region and a relatively homogenous desert climate.

## 2 MATERIALS AND METHODS

### 2.1 Study area

**FIGURE 1:**
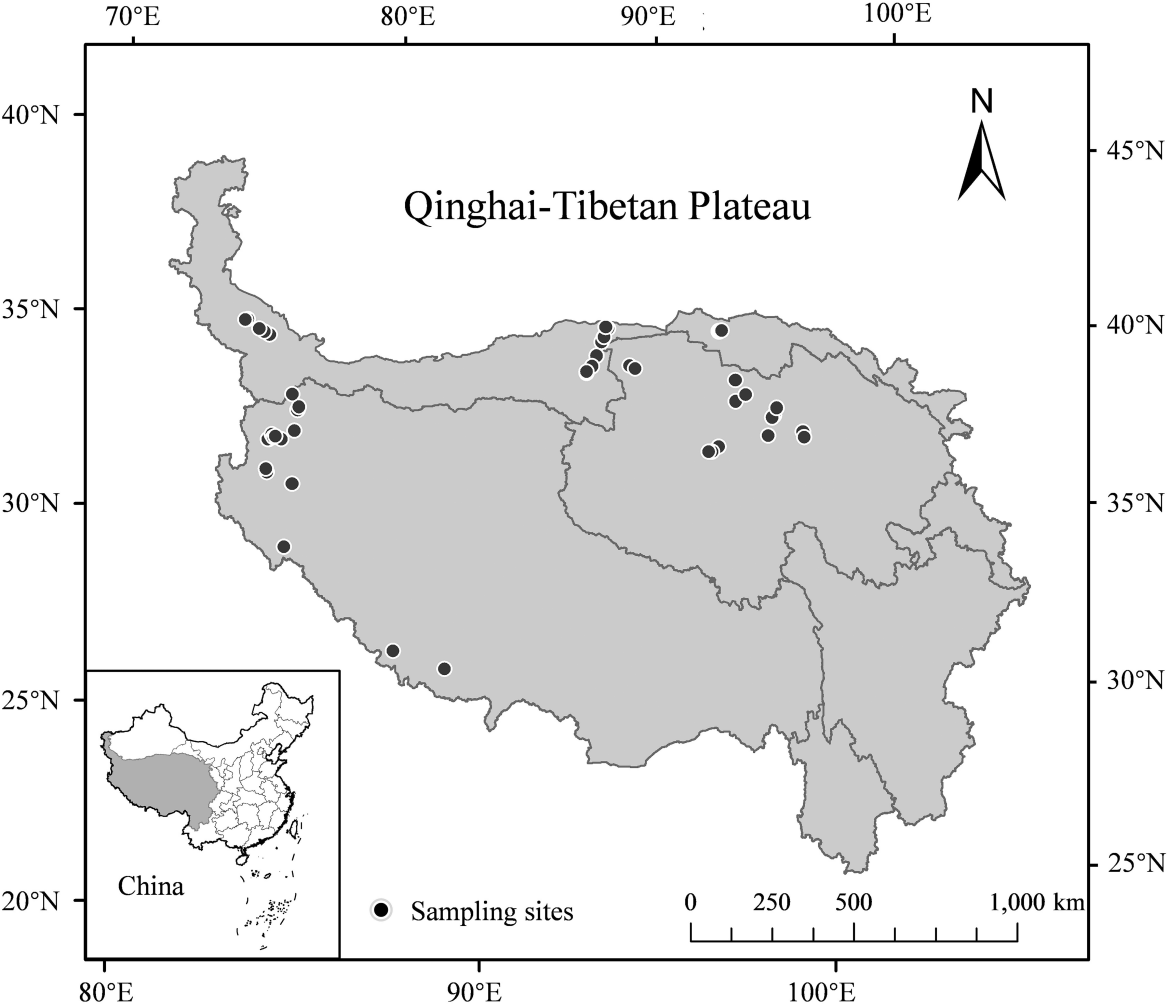
Distribution of sample points in the Qinghai–Tibet Plateau desert.

The study area is in the northeastern, northwestern, and southwestern parts of the QTP, mainly including parts of Gansu Province, Qinghai Province, Xinjiang Autonomous Region, and Tibet Autonomous Region (Figure 1). The altitude ranges from 2659 to 5197 m. The temperature changes dramatically throughout the year, with the highest temperature being 18.5°C and the lowest temperature being −20.6°C, with an average temperature of −0.04°C. Precipitation is scarce, with an annual average of 85 mm. The vegetation in the study area is mainly composed of drought‐tolerant and ultradrought‐tolerant shrubs, semishrubs, and small trees, as well as drought‐tolerant herbs (Wang, Wang, et al., 2022b). Dominant species in the shrub layer include *Sympegma regelii*, *Salsola abrotanoides*, *Ceratoides compacta*, *Ceratoides latens*, *Reaumuria soongorica*, *Haloxylon ammodendron*, and *Ajania fruticulosa*.

### 2.2 Field survey

From 2017 to 2019, 53,100 m × 100 m plots were surveyed. The plots were strategically selected based on the spatial distribution of the desert ecosystem and were conducted as comprehensive plant community surveys across diverse vegetation types. A single 10 m × 10 m quadrat was used in each plot. Among these plots, two did not have shrubs, and 18 did not have herb plants. At each site, we used a global positioning system (GPS) device to obtain the location (longitude, latitude, and altitude) of the plot and recorded the species names, mean height, and cover of herb and shrub species in each plot.

### 2.3 Data sources

#### 2.3.1 Environmental distance

Environmental distance was defined as the differences in climatic factors, soil properties, and altitude between sampling sites. Climatic factors included mean annual precipitation (MAP), mean annual temperature (MAT), mean temperature of the coldest month (MTCM), mean temperature of the warmest month (MTWM), potential evapotranspiration (PET), relative humidity (RH), and wind speed (WND). Climatic data were obtained from the National Earth System Science Data Sharing Service Platform and extracted using ArcGIS 10.2 (Jing et al., 2016; Peng et al., 2019). Altitude (ALT) was measured on‐site during sampling. Soil factors, included soil total nitrogen content (TN), total phosphorus (TP), total potassium (TK), pH, soil organic carbon content (SOC), available nitrogen (AN), available phosphorus (AP), available potassium (AK), and gravel coverage (GC), were derived from spatial data with a precision of 90 m at a soil depth of 0–20 cm using ArcGIS 10.2 (Liu et al., 2020, 2022). All environmental factors were standardized for subsequent analyses.

#### 2.3.2 Geographic distance

Geographical distance refers to the distance between sampling plots, which is determined by the following two formulas:

(1) $$\begin{array}{c} D=R\times \text{Arccos}[\sin\left(90^{{}^{\circ}}-\text{LatA}\right)\times \sin\left(90^{{}^{\circ}}-\text{LatB}\right)\times \cos\left(\text{LonA}-\text{LonB}\right)\\ +\cos\left(90^{{}^{\circ}}-\text{LatA}\right)\times \cos\left(90^{{}^{\circ}}-\text{LatB}\right)]\times \mathrm{Pi}/180 \end{array}$$

where *D* is the geographical distance between the communities A and B, *R* is the radius of the Earth (6371.004 km), LatA and LatB represent the latitudes of the communities A and B, LonA and LonB represent the longitudes of the communities A and B, and Pi is the value of π. To facilitate subsequent analysis, the adeSpatial package (Dray et al., 2017) dbmem function in R 4.1.3 was used to calculate distance‐based Moran eigenvector maps (dbMEM, also known as dbMEMspatial eigenfunctions) from the geographical distance matrix, which were used for spatial feature function analysis to create spatial feature variables.

#### 2.3.3 Plant functional traits

This study selected plant height (H, cm), leaf area (LA, cm^2^), leaf thickness (LT, mm), leaf dry matter content (LDMC, g·g^−1^), and specific leaf area (SLA, cm^2^·g^−1^) five plant functional traits. Plant trait data were subjected to principal component analysis (PCA) using the prcomp function to extract the first two principal components (Table S1). The resulting components were employed for the analysis of functional beta diversity. H was measured by selecting 15 healthy and pest‐free individuals during field surveys. LA was calculated using species‐specific leaf length, width, and shape data obtained from the Flora of China. LT, LDMC, and SLA were obtained through the interrogation of the TRY database (Kattge et al., 2020) and relevant published literature (Jin et al., 2023).

### 2.4 Calculation and partitioning of beta diversity

In this study, the Jaccard community similarity coefficient based on species abundance was used (Baselga, 2010). The Jaccard community similarity coefficient (*β*_jac_) was decomposed into spatial turnover components (*β*_jtu_) and nestedness components (*β*_jne_) (Baselga, 2012). The specific calculation formula is as follows:

(2) $$\beta_{\mathrm{jtu}}=2\times \min\left(b,c\right)/\left(a+2\times \min\left(b,c\right)\right)$$

(3) $$\begin{array}{c} \beta_{\mathrm{jne}}=\left|b+c\right|/\left(a+b+c\right)\times a/\left(a+2\times \min\left(b,c\right)\right)\; \end{array}$$

(4) $$\begin{array}{c} \beta_{\mathrm{jac}}=\left(b+c\right)/\left(a+b+c\right)=\beta_{\mathrm{jtu}}+\beta_{\mathrm{jne}} \end{array}$$

where *a* represents the number of shared species between the paired sample plots, and *b* and *c* are the numbers of unique species in each plot. The proportions of species turnover and nestedness components are represented by *β*_jtu_/*β*_jac_ and *β*_jne_/*β*_jac_, respectively, to analyze which component dominates.

The community taxonomic, phylogenetic, and functional *β* diversity, as well as their components, were calculated using the beta.pair, phylo.beta.pair, and func.beta.pair functions, respectively, in the R package *betapart* (Baselga & Orme, 2012). The community phylogenetic *β* diversity was calculated based on the phylogenetic tree, which was constructed using the phylo.maker function in R package *V.PhyloMaker* (Jin & Qian, 2019). The community functional *β* diversity was calculated using Euclidean distances between two selected plant functional traits (Villeger et al., 2013). Due to the absence of shrubs or herbs, the number of plots available for analyzing *β* diversity in the shrub layer was 52, and that for the herb layer was 35. Furthermore, in half of the plots, the species number of shrubs communities was lower than the number of functional traits we selected. Therefore, we did not separately calculate the functional *β* diversity of the herb and shrub layers.

### 2.5 Statistical analysis

We used Mantel tests within the *vegan* package (Oksanen et al., 2017) to analyze whether there was a significant correlation between geographic and environmental distances and plant community taxonomic, phylogenetic, and functional beta diversity and their decomposition components. Variance partitioning analysis (VPA) was conducted to assess the relative contributions of environmental factors (including altitude, climate factors, and soil factors) and geographic distance to the three facets of beta diversity of plant communities. This analysis was performed using the varpart function within the *vegan* package. Additionally, we employed the *betapart* package (Baselga, 2012) to conduct distance‐based redundancy analysis (db‐RDA) in order to identify the environmental factors influencing plant community beta diversity. Cluster analysis was performed using the varclus function in the *Hmisc* package (Harrell & Dupont, 2018) to avoid the impact of multicollinearity. Variables with high correlation (Spearman ρ^2^ > .5) were removed from the analysis (Figure S1). Finally, three climate factors (mean temperature of the coldest month, mean annual precipitation, and mean wind speed), six soil factors (total phosphorus, total potassium, soil pH, available nitrogen, available potassium, gravel cover), and altitude were used together for distance‐based redundancy analysis. Data processing and related figure production were completed in R4.1.3 using *vegan* (Oksanen et al., 2017), *adeSpatial* (Dray et al., 2017), *V.PhyloMaker* (Jin & Qian, 2019), *betapart* (Baselga, 2012), *Hmisc* (Harrell & Dupont, 2018), and *ggplot2* (Wickham, 2009) packages.

## 3 RESULTS

### 3.1 Beta diversity of plant communities and their decomposition components

**FIGURE 2:**
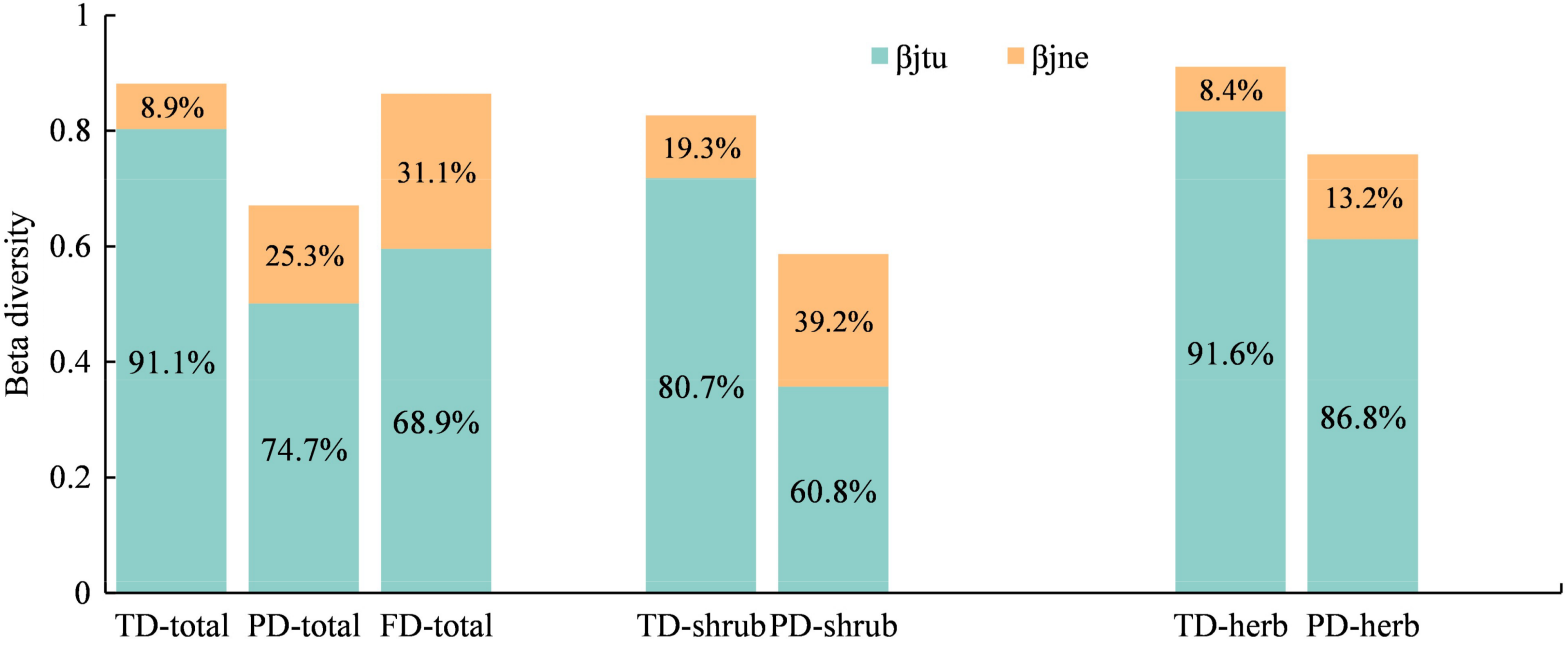
Taxonomic, phylogenetic, and functional beta diversity and their compositions in the QTP desert. *β*_jne_, nestedness component; *β*_jtu_, turnover component; FD, functional diversity; PD, phylogenetic diversity; TD, taxonomic diversity. The percentages in the figure represented the relative proportion of turnover and nestedness components.

A total of 62 plant species were recorded, including 26 shrubs and 36 herbs, with herbs accounting for 58.07% of the total species number. For the whole community, taxonomic beta diversity in the QTP desert (0.88) was dominated by turnover (0.80), with only a small contribution from nestedness (0.08) (Figure 2). With minor variations, similar patterns were found for phylogenetic and functional beta diversity, and for the taxonomic and phylogenetic beta diversity components for shrubs and herbs separately.

### 3.2 Relationships between environmental and geographic distances and *β* diversity

Mantel tests indicated that both environmental and geographic distances were significantly related to taxonomic, phylogenetic, and functional beta diversity for the whole community (*p* < .05) (Table 1). Similar patterns were found for the turnover components of the three facets of beta diversity and for the taxonomic and phylogenetic beta diversity for shrubs and herbs.

**TABLE 1:** Mantel tests for the relationships between environmental distance, geographic distance and the taxonomic, phylogenetic, and functional beta diversity of different growth forms.

Beta Jaccard	Effect of	Total			Shrub			Herb		
		*β*_jac_	*β*_jtu_	*β*_jne_	*β*_jac_	*β*_jtu_	*β*_jne_	*β*_jac_	*β*_jtu_	*β*_jne_
		*r*	*r*	*r*	*r*	*r*	*r*	*r*	*r*	*r*
Species	env	.420***	.357***	−.2	.318***	.315***	−.34	.514***	.488***	−.21
	geo	.425***	.381***	−.234	.299***	.299***	−.3	.465***	.438***	−.203
Phylogenetic	env	.339***	.357***	−.157	.362***	.322***	−.063	.288**	.279*	−.175
	geo	.374***	.405***	−.234	.295***	.285***	−.083	.275**	.272**	−.178
Functional	env	.032	.054	−.046						
	geo	.094*	.157***	−.132						

Abbreviations: *β*_jac_, beta diversity; *β*_jne_, nestedness component of beta diversity; *β*_jtu_, turnover component of beta diversity; env, environmental distance; geo, geographic distance.

*** *p* < .0001.

** *p* < .001.

* *p* < .05.

**FIGURE 3:**
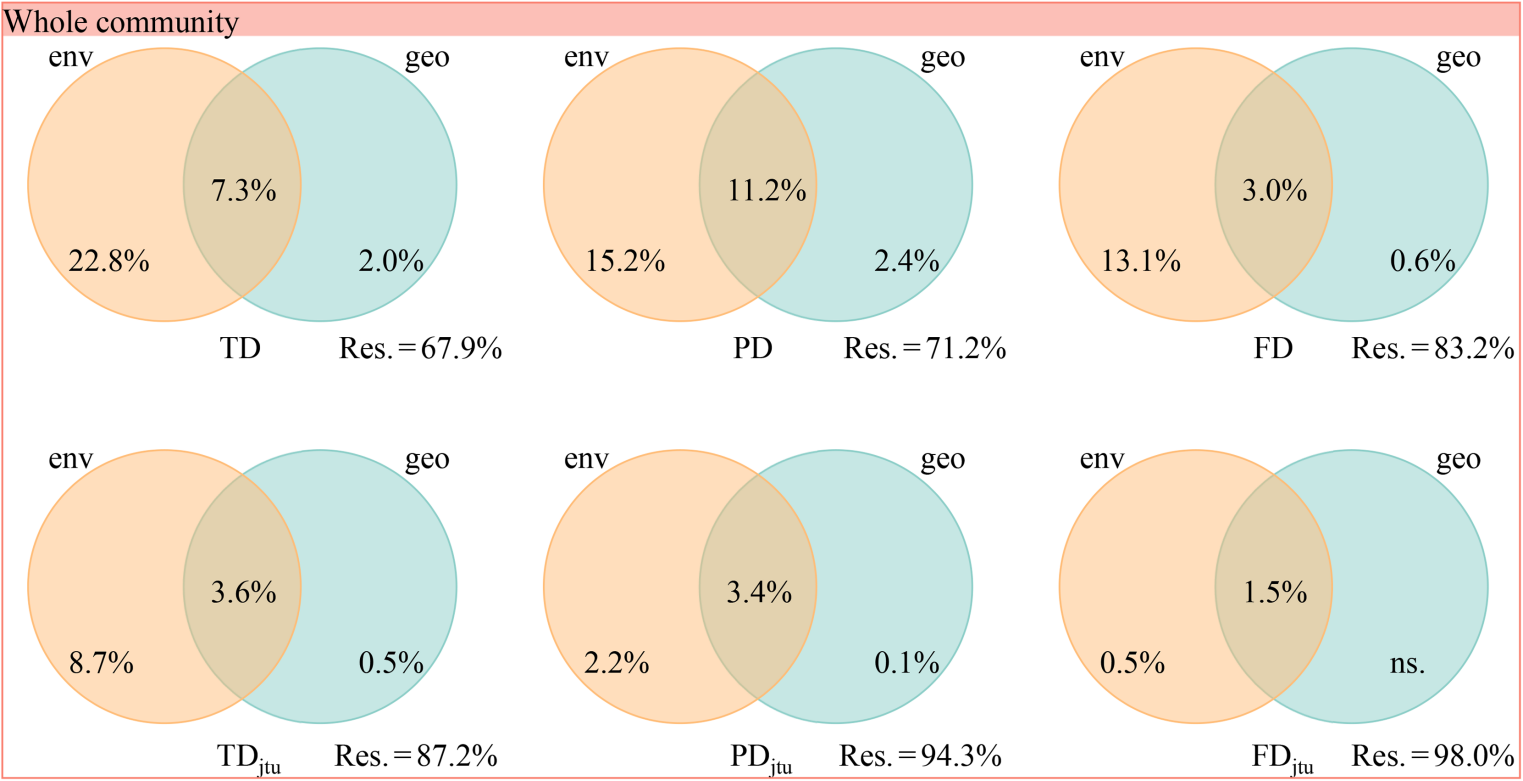
Variation partitioning for the relative influence of environmental and geographical distances on taxonomic, functional, and phylogenetic beta diversity and their turnover components for the whole community in the QTP desert. env, environmental distance; FD, functional diversity; FD_jtu_, turnover component of functional diversity; geo, geographic distance; PD, phylogenetic diversity; PD_jtu_, turnover component of phylogenetic diversity; TD, taxonomic diversity; TD_jtu_, turnover component of taxonomic diversity.

**FIGURE 4:**
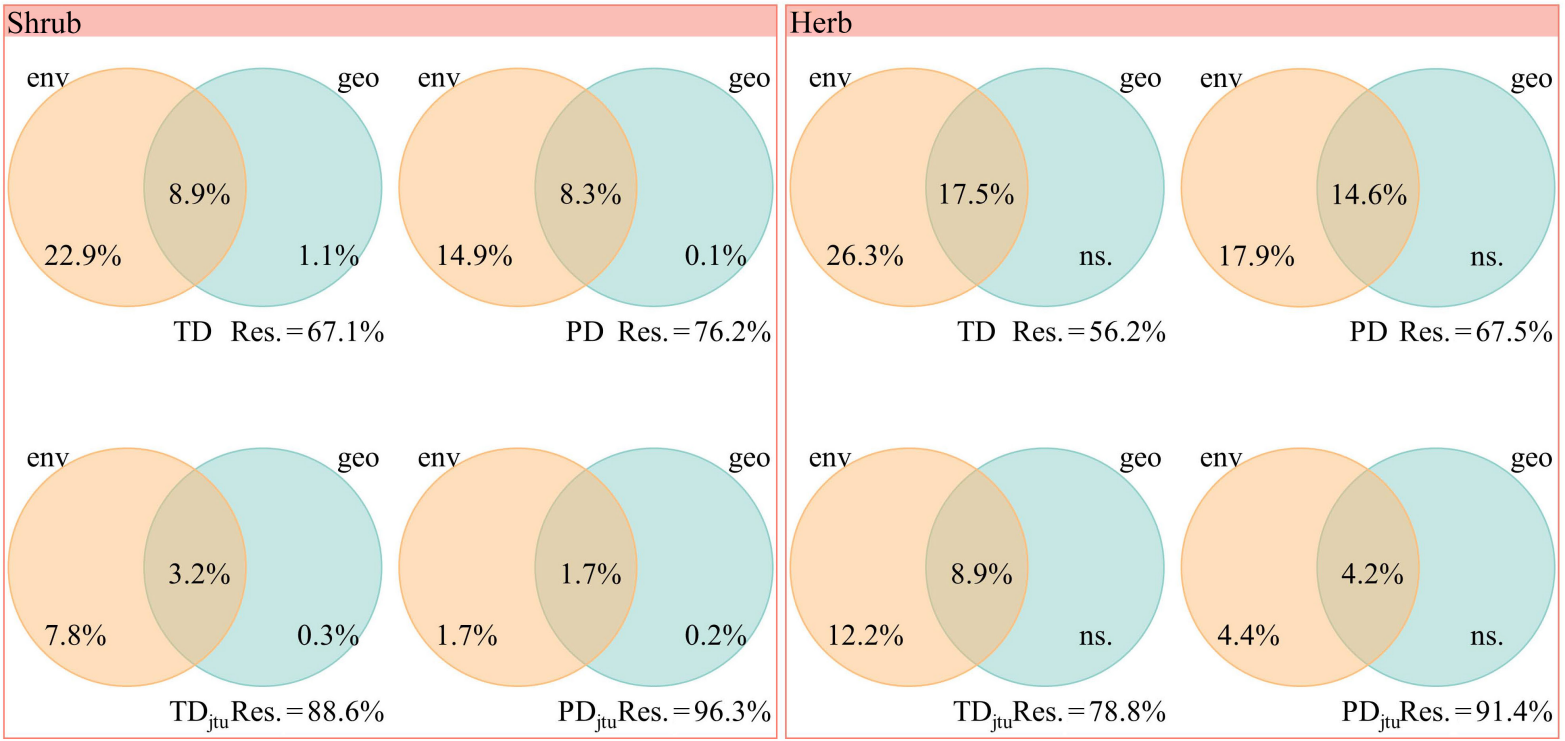
Variation partitioning for the relative influence of environmental and geographical distances on taxonomic and phylogenetic beta diversity and their turnover components of herb and shrub in the QTP desert. env, environmental distance; geo, geographic distance; PD, phylogenetic diversity; PD_jtu_, turnover component of phylogenetic diversity; TD, taxonomic diversity; TD_jtu_, turnover component of taxonomic diversity.

For the whole community, environmental distance explained 22.8%, 15.2%, and 13.1% of the variation in taxonomic, phylogenetic, and functional beta diversity, respectively (Figure 3). In contrast, geographic distance explained only 2.0%, 2.4%, and 0.6% of the variation in the three facets of beta diversity. Environmental distance also played a dominant role in shaping the turnover components of the three facets of beta diversity for the whole, herb, and shrub communities (Figures 3 and 4), which supported H2.

### 3.3 Impacts of key environmental factors on the beta diversity of plant communities

**FIGURE 5:**
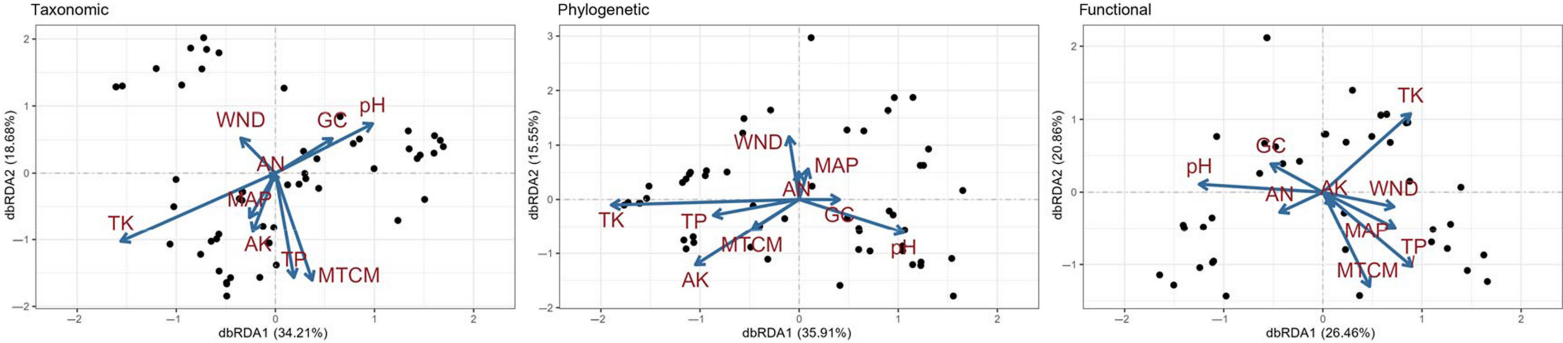
Distance‐based redundancy analyses (db‐RDA) of taxonomic, phylogenetic, and functional beta diversity in relation to key variables. MAP represents mean annual precipitation. MTCM represents mean temperature of the coldest month. PET represents potential evapotranspiration. RH represents relative humidity. TP represents soil total phosphorus content. TK represents soil total potassium content. AK represents soil available potassium content. GC represents the gravel coverage.

Among the considered environmental factors, both climatic and soil factors exhibited the highest explanatory power in relation to beta diversity and turnover components across the whole community, as well as within the shrub and herb communities (Table 2). Altitude had a smaller influence than climatic and soil factors, with the former only uniquely explaining 0%–2.1% of beta diversity. The db‐RDA results revealed that, for the whole community, soil total potassium content and soil pH exerted the most significant influence on all three facets of beta diversity (Figure 5). Additionally, both the mean temperature of the coldest month and soil total phosphorus content had significant impacts on both taxonomic and functional beta diversity. For phylogenetic beta diversity, available potassium also had substantial impacts.

**TABLE 2:** Variation partitioning for the relative influence of altitude, climatic, and soil factors on taxonomic, phylogenetic, and functional beta diversity and their turnover components in the QTP desert.

	Beta Jaccard	Clim	Soil	Alt	Clim + soil	Clim + alt	Soil + alt	Clim + soil + alt
Total	TD	10.1%	7.9%	1.0%	4.9%	0.8%	4.9%	1.2%
	PD	7.3%	8.4%	0.0%	1.4%	0.6%	8.7%	7.0%
	FD	7.4%	8.3%	0.0%	ns.	0.0%	0.3%	1.5%
	TD_jtu_	4.2%	3.0%	0.6%	2.2%	0.2%	1.8%	0.3%
	PD_jtu_	0.9%	1.7%	ns.	0.4%	0.2%	0.6%	0.3%
	FD_jtu_	0.1%	1.1%	0.0%	0.2%	0.0%	ns.	0.8%
Shrub	TD	9.8%	7.8%	0.7%	7.0%	2.8%	3.7%	0.1%
	PD	3.6%	5.3%	ns.	7.4%	2.4%	2.5%	2.1%
	TD_jtu_	3.5%	2.2%	0.3%	2.6%	0.8%	1.4%	0.2%
	PD_jtu_	ns.	0.6%	ns.	1.5%	0.3%	0.3%	0.7%
Herb	TD	9.3%	9.7%	1.7%	11.5%	2.2%	8.7%	0.6%
	PD	6.6%	7.5%	1.8%	4.5%	3.7%	3.0%	5.2%
	TD_jtu_	3.9%	4.4%	0.7%	6.2%	1.0%	4.5%	0.5%
	PD_jtu_	1.4%	2.7%	ns.	0.4%	1.2%	1.0%	1.8%

Abbreviations: alt, altitude; clim, climate factors; FD, functional beta diversity; FD_jtu_, turnover component of functional beta diversity; PD, phylogenetic beta diversity; PD_jtu_, turnover component of phylogenetic beta diversity; soil, soil factors; TD, taxonomic beta diversity; TD_jtu_, turnover component of taxonomic beta diversity.

## 4 DISCUSSION

### 4.1 The components of plant community beta diversity

The turnover component played a vital role in shaping the taxonomic, phylogenetic, and functional beta diversity of the plant community in the QTP desert across all plants as well as shrubs and herbs separately. Similar patterns have also been observed in previous studies (Piroozi et al., 2018; Soininen et al., 2018; Wang et al., 2018). As hypothesized in H1, the turnover component exhibited lower values in both phylogenetic and functional beta diversity compared to taxonomic beta diversity. For phylogenetic beta diversity, the pattern may be attributed to the fact that the glacier area of the QTP during the last glacial period was only slightly larger than that of modern glaciers (Kirchner et al., 2011; Křížek & Mida, 2013). The relatively small difference in glacier area might have resulted in high survival rates among the resident species in the region (Colville et al., 2020; D'Souza & Hebert, 2018; Qian et al., 2020; Svenning et al., 2011), leading to the lower ratio of turnover components in phylogenetic beta diversity, that is, because population extirpations have not prohibited local diversification and species from locally diversified clades have had high survival rate (Dynesius & Jansson, 2000). Furthermore, the smaller turnover component in functional beta diversity may due to the trait convergence. In this region, the unique environmental conditions are characterized by low temperatures, strong ultraviolet radiation stress, low oxygen pressure, and low accumulated temperature (Rocha et al., 2021; Zheng & Zhao, 2019), which may have led to the formation of consistent adaptation strategies and species coexistence mechanisms for plants to cold, dry climates (Butterfield & Briggs, 2011; Huang et al., 2016).

H1 is also supported by the results of herbs and shrubs. However, comparing taxonomic and phylogenetic beta diversity, herbs consistently had higher beta diversity than shrubs. This may be due to higher sensitivity to environmental resource gradients or specific habitat types of herbs compared to shrubs in arid desert ecosystems (Murphy et al., 2016; Wang et al., 2021). Previous work on arid ecosystem indicated that herbs were highly sensitive to water and nutrient availability (Cui et al., 2017; Karatas, 2019; Le Houérou et al., 1988; Yahdjian & Sala, 2006). Especially, perennial herbs in desert region exhibit lower tolerance to environmental change and are more likely to be facilitated by neighbors than shrubs (Bai et al., 2019). Additionally, herbs have proportionally more species with dormant seeds compared to shrubs (Jurado & Flores, 2005), and exhibit highly specific and variable germination requirements (Commander et al., 2017; Murphy et al., 2016). Besides, due to the short stature and small seed size, which restricts animal dispersal, herbs are highly dispersal limited (Hughes et al., 1994).

### 4.2 Influences of environmental and geographic distances

Our results support the second hypothesis (H2), indicating that environmental filtering and dispersal limitation jointly determine beta diversity in the QTP desert, with environmental filtering played a dominant role. These results are consistent with previous observations in various ecosystems (Gravel et al., 2006; Hu et al., 2022; Jiang et al., 2022; Mori et al., 2018). The high altitude variation, reaching up to 2538 m, further contributes to substantial heterogeneity in environmental conditions. While altitude is a mainly indirect determinant of the spatial distribution of plant species (Guisan & Zimmermann, 2000; Svenning et al., 2009), it can affect heterogeneity in other environmental factors and increase the spatial isolation of species (Qian & Ricklefs, 2012). This study revealed that altitude had a relatively low individual explanatory power, but a higher co‐explanatory power when considered alongside environmental factors. The results of dbRDA indicated that, concerning taxonomic beta diversity, plots at elevations above 4000 m were primarily distributed on the right side of the y‐axis, which plots below 4000 m were mainly distributed on the left side. This may be because changes in altitude lead to changes in temperature as well as sometimes other environmental factors such as precipitation, light, and soil, which often involve differences in habitat water and heat (Tang et al., 2012). Higher environmental heterogeneity promotes environmental filtering playing a major role in shaping the observed beta diversity (Legendre et al., 2009; Page & Shanker, 2018). Furthermore, altitude plays a crucial role in the distribution of glaciers during maximum glaciation (Křížek & Mida, 2013). The climate during the Plio‐Pleistocene period shaped the current patterns of plant diversity and the relationship with the environment in the QTP (Li et al., 2013; Wen et al., 2014). In Europe, regions without glaciers during the Plio‐Pleistocene period exhibit higher plant diversity and stronger environmental relationships than regions that were glaciated (Svenning et al., 2009). These findings indicated that the absence of glaciers during Plio‐Pleistocene had contributed to the plant diversity and environmental relationships.

Although environmental filtering played a dominant role in shaping beta diversity, its importance in herbs was higher compared to shrubs, consistent with the previous finding that herbs were more sensitive to environmental conditions (Murphy et al., 2016; Tang et al., 2012; Wang et al., 2021). The larger individual and seed sizes of shrub species compared to herb species make them more adapted for dispersal by vertebrates (Hughes et al., 1994), which in turn leads to a stronger correlation between shrub plant community beta diversity and geographic distance.

### 4.3 Impact of soil and climate factors

In alignment with H3, soil factors exhibited a greater explanatory power in shaping three facets of beta diversity and the turnover components. This is consistent with previous studies in the QTP, emphasizing the critical role of soil factors in governing plant community dynamics (Ade et al., 2021; Wang et al., 2023). Notably, among soil factors, soil potassium, phosphorus content, and pH emerged as primary influencers on the three facets of beta diversity in this region. In arid environments, soil potassium regulates the leaf and root nitrogen content, impacting plants' nutrients acquisition strategies (Luo et al., 2021). Sufficient potassium can enhance plant dry matter and root surface area, leading to improved water absorption and aiding plants' ability to cope with drought (Egilla et al., 2001). Furthermore, under cold or freezing stress, potassium can regulate the osmotic potential of plants, thereby reducing dehydration caused by freezing (Wang et al., 2013) and preventing frost damage (Fazeli et al., 2007). In this study, the difference between *Ajania fruticulosa*‐dominated assemblages and other communities showed negatively correlated with soil total potassium content. Conversely, the *Salsola abrotanoides* community, primarily situated on the silt desert in front of the alpine region, exhibited positive correlation with soil total potassium content. Similarly, soil phosphorus, essential for photosynthesis and water use efficiency (He et al., 2016), influences plant survival (Huang et al., 2018; James et al., 2005). The dissimilarity of the *Sympegma regelii* community exhibited a positive correlation with soil total phosphorus, while the dissimilarity of the *Ceratoides compacta* community, which has a relatively narrow distribution range, being confined to slopes and gravel areas at elevations of 3500–5000 m, showed a negatively correlated with soil total phosphorus.

While the impact of climate factors on beta diversity in the QTP desert is smaller compared to soil factors, the results underscored the substantial influence of the mean temperature of the coldest month on both taxonomic and functional beta diversity. This pattern aligns with previous findings in QTP forests (Zhang et al., 2023). In alpine environments, sets limits on species' fundamental niches and affects ecological processes like decomposition, nutrient cycling, and water availability (Graae et al., 2018). In the QTP, the primary constraints on plant growth arise from the combination of low temperatures and the resulting shorter growing seasons (Zhang et al., 2015). The negative correlation between *Ceratoides compacta* communities and the mean temperature of the coldest month aligns with its distribution in extremely cold and dry climates, providing further evidence of the temperature effect observed in this study.

## 5 CONCLUSION

Investigating the effects of environmental and geographic distances on plant beta diversity and the mechanisms that determine these patterns in the QTP desert is vital for our understanding of the basis for the region's plant diversity. Generally, this study indicates that species turnover dominated the taxonomic, phylogenetic, and functional beta diversity of plant communities. The turnover component in phylogenetic and functional beta diversity was smaller compared to that of taxonomic beta diversity, consistent with likely high rates of local diversification and glacial survival as well as convergent trait selection due to the extreme environment. Although both environmental and geographic distances were significantly correlated with beta diversity and the turnover component, environmental factors had stronger correlations. Our findings strongly indicate that environmental sorting plays a major role in determining the composition of plant communities in the QTP desert ecosystem. Among the environmental factors, soil potassium was a significant predictor of taxonomic, phylogenetic, and functional beta diversity. Moreover, the mean temperature of the coldest month and soil total phosphorus strong correlated with taxonomic and functional beta diversity. Our findings indicate that incorporating geodiversity mapping and assessment into conservation planning will be important for the long‐term protection of vegetation diversity in the QTP desert.

## AUTHOR CONTRIBUTIONS

**Lu Wen:** Conceptualization (lead); formal analysis (lead); funding acquisition (lead); investigation (lead); supervision (lead); writing – original draft (lead); writing – review and editing (lead). **Kexuan Zhao:** Data curation (lead); formal analysis (lead); writing – original draft (lead). **Haoyu Sun:** Writing – review and editing (equal). **Gang Feng:** Formal analysis (equal); writing – review and editing (supporting). **Qiang Sun:** Data curation (supporting); investigation (equal). **Cunzhu Liang:** Investigation (equal); writing – review and editing (supporting). **Zhiyong Li:** Investigation (equal); writing – review and editing (supporting). **Lixin Wang:** Supervision (supporting); writing – review and editing (supporting). **Jens‐Christian Svenning:** Supervision (supporting); writing – original draft (supporting); writing – review and editing (equal).

## FUNDING INFORMATION

This work was supported by grants from the Second Tibetan Plateau Scientific Expedition and Research Program (STEP) (grant 2019QZKK0307), the National Natural Science Foundation of China (grant 31960249, 32160279) and the Science and Technology Program of Inner Mongolia Autonomous Region of China (grant 2022YFHH0024, 2022YFHH0017, 2021GG0307). JCS considers this work a contribution to Centre for Ecological Dynamics in a Novel Biosphere (ECONOVO), funded by Danish National Research Foundation (grant DNRF173) and his VILLUM Investigator project “Biodiversity Dynamics in a Changing World”, funded by VILLUM FONDEN (grant 16549).

## CONFLICT OF INTEREST STATEMENT

The authors declare that they have no conflict of interest.

## Supporting information


Figure S1.

Figure S2.

Table S1.

Appendix S1.

## Data Availability

The data that support the findings of this study are available within the supplementary materials.
