# Supplementary figures and images for: Drivers of desert plant beta diversity on the Qinghai–Tibet plateau
Source: Ecol Evol. 2024 Feb 20;14(2):e10993. doi: 10.1002/ece3.10993 (PMC10877311; doi:10.1002/ece3.10993)

Spearman  $\rho^z$

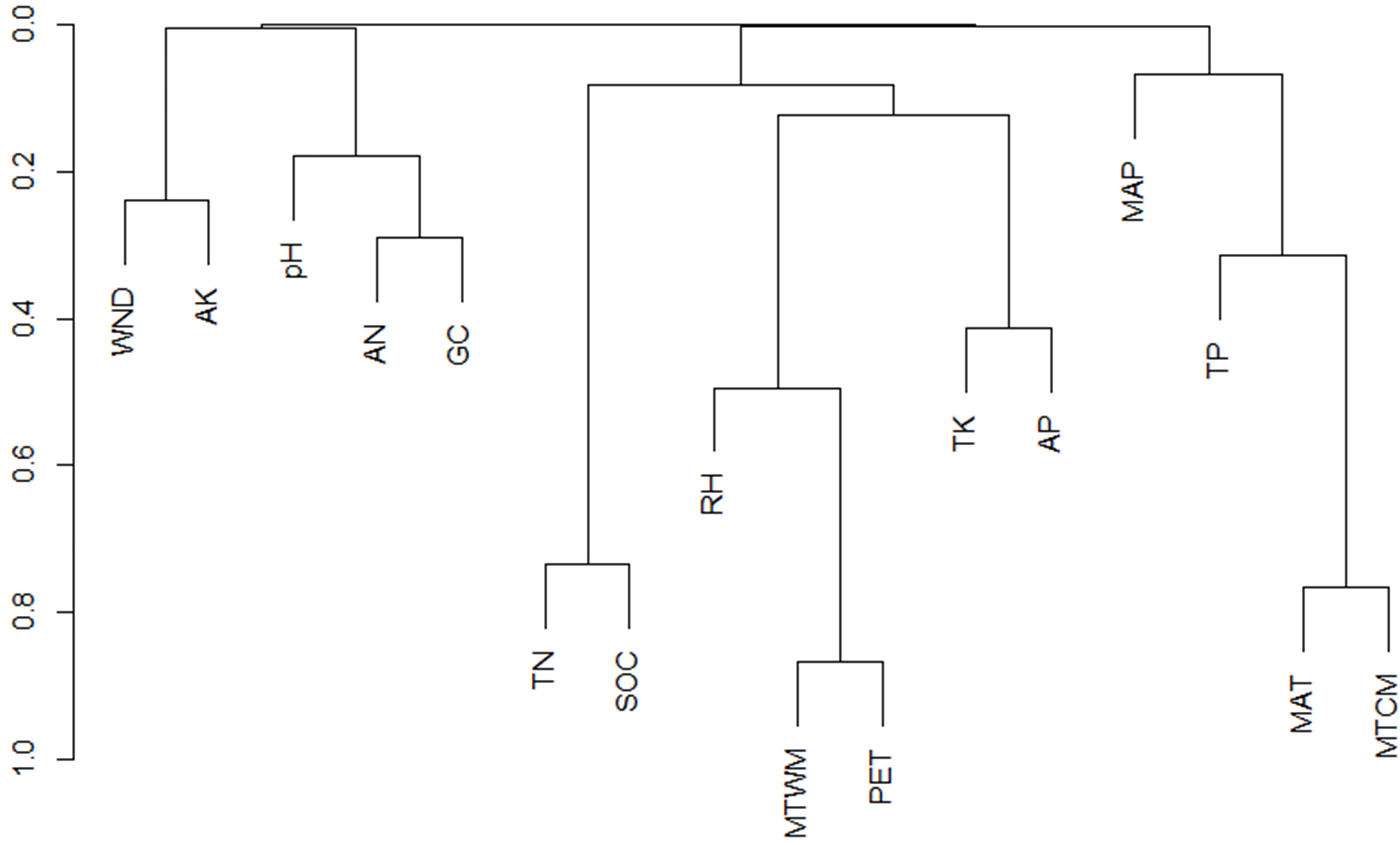

Supplement: Supplementary file 1 — Figure S1. [file ECE3-14-e10993-s001.pdf]

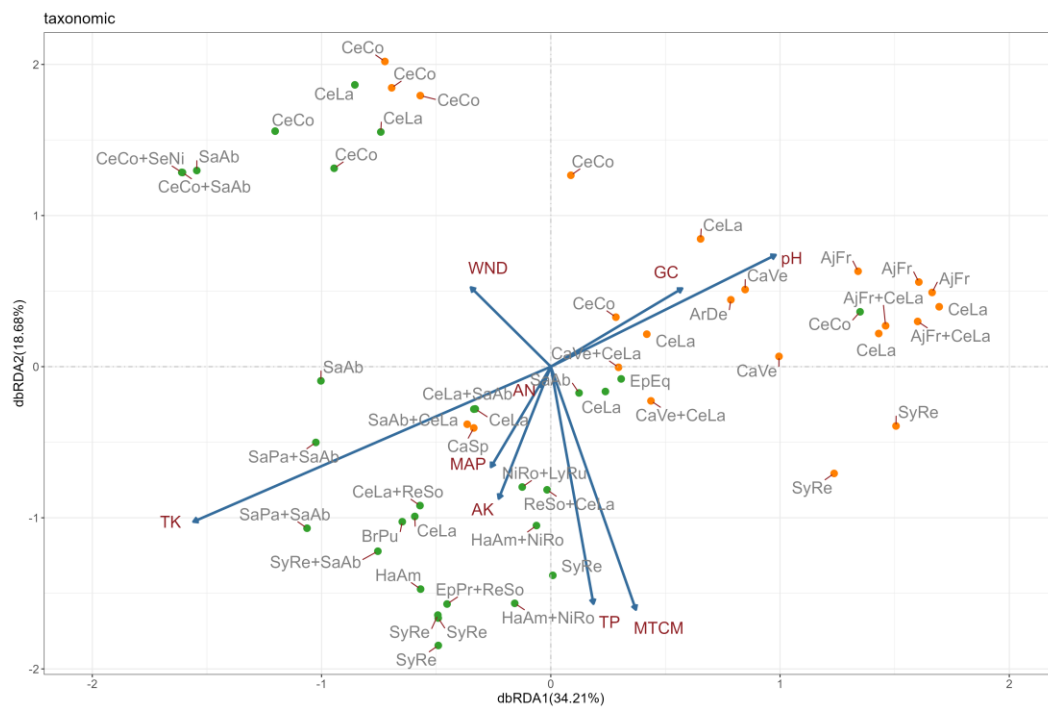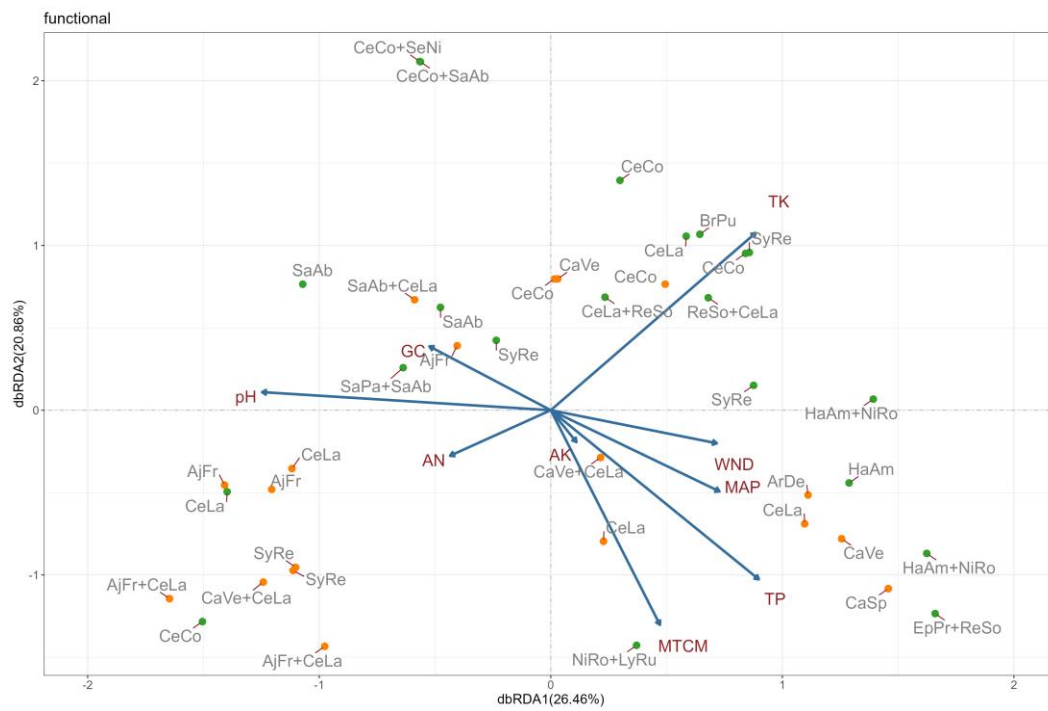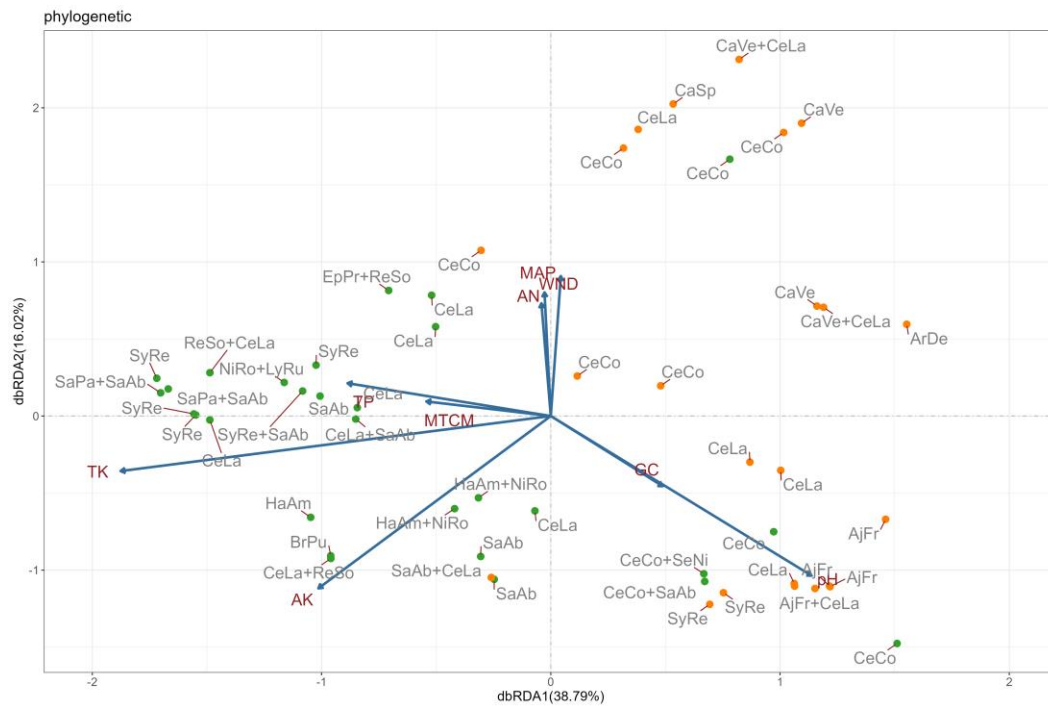

Supplement: Supplementary file 2 — Figure S2. [file ECE3-14-e10993-s004.pdf]
