# Supplementary material for: Drivers of desert plant beta diversity on the Qinghai–Tibet plateau
Source: Ecol Evol. 2024 Feb 20;14(2):e10993. doi: 10.1002/ece3.10993 (PMC10877311; doi:10.1002/ece3.10993)
Supplement: Supplementary file 3 — Table S1. [file ECE3-14-e10993-s002.docx]

Table S1 Results of the principal component analysis for five species traits

| Traits | Loading on each factor in the PCA | |
| --- | --- | --- |
|  | PC1 | PC2 |
| SLA | 0.613 | 0.396 |
| LT | 0.326 | -0.639 |
| LDMC | -0.679 | -0.094 |
| H | 0.022 | 0.467 |
| LA | -0.239 | 0.455 |

PC1 and PC2 represent the first and second principal components, respectively, PC1 and PC2 can explain 27.7% and 24.0% of the variation in five traits, respecitvely. SLA represents specific leaf area, LT represents leaf thickness, LDMC represents leaf dry matter content, H represents height, LA represent leaf area.
